# Promoting psychological wellbeing in hospitality: the interactive roles of autonomy-supportive leadership, psychological need satisfaction, and perceived organizational support

**DOI:** 10.3389/fpsyg.2026.1813978

**Published:** 2026-04-22

**Authors:** Ahmed Hassan Abdou, Abdullah Saleh Mohammed Albohnayh

**Affiliations:** 1 Social Studies Department, College of Arts, King Faisal University, Al-Ahsa, Saudi Arabia; 2 Department of Education and Psychology, College of Education, King Faisal University, Al-Ahsa, Saudi Arabia

**Keywords:** hospitality industry, need satisfaction, organizational support, Saudi Arabia, Self-Determination Theory, S–O–R model, supportive leadership

## Abstract

**Introduction:**

Despite growing interest in leadership and employee wellbeing, limited research has empirically examined how autonomy-supportive leadership (ASL) enhances psychological wellbeing (PWB) in high-demand hospitality settings, particularly regarding the underlying mechanisms and boundary conditions. This study addresses this gap by examining the mediating role of basic psychological need satisfaction (PNS; autonomy, competence, and relatedness) and the moderating role of perceived organizational support (POS).

**Methods:**

Grounded in Self-Determination Theory (SDT), Organizational Support Theory, and the Stimulus–Organism–Response (S–O–R) framework, this study employed a three-wave, time-lagged design. Data were collected from 394 frontline employees working in five-star hotels in Saudi Arabia. The proposed model was tested using partial least squares structural equation modeling (PLS-SEM) with bootstrapping to assess direct, indirect, moderating, and moderated mediation effects.

**Results:**

The findings reveal that ASL significantly and positively predicts both PWB (β = 0.504, *p* < 0.001) and PNS (β = 0.455, *p* < 0.001). PNS partially mediates the relationship between ASL and PWB (indirect effect β = 0.098, 95% CI [0.045, 0.149]). Additionally, POS strengthens the positive effects of ASL on PNS (β = 0.107, *p* < 0.05) and on PWB (β = 0.133, *p* < 0.001) and moderates the indirect effect of ASL on PWB via PNS (β = 0.023, *p* < 0.05).

**Discussion:**

By integrating leadership style and organizational context into a moderated mediation framework, this study provides robust empirical evidence that combining ASL with strong organizational support enhances employee wellbeing. These findings offer practical implications for fostering healthier and more sustainable work environments in high-pressure hospitality settings.

## 1 Introduction

The hospitality industry is recognized as one of the most psychologically demanding service sectors (Abdou et al., 2024). Frontline employees face high work pressure, emotional labor, irregular schedules, and strict service standards, all while maintaining positive interactions with guests. These challenges often lead to stress, burnout, and impaired psychological functioning (Abolnasser et al., 2023; Salama et al., 2022). Consequently, understanding how to support employees' psychological wellbeing (PWB) is critical for both research and practice, given its direct impact on service quality, staff retention, and organizational performance (Wong et al., 2025; Walbeek and El Hajal, 2022).

Leadership plays a pivotal role in shaping employees' experiences at work. In hospitality, supervisors translate organizational expectations into daily practices, directly influencing employee engagement and wellbeing (Abolnasser et al., 2023; Zia et al., 2022). Prior research shows that supportive leadership improves job satisfaction, engagement, and reduces burnout (Abolnasser et al., 2023; Lin and Ling, 2021; Ozturk et al., 2021). However, most studies focus on general leadership styles and direct effects, leaving the mechanisms and boundary conditions linking leadership to PWB largely unexplored. Specifically, the role of autonomy-supportive leadership (ASL), which emphasizes listening to employees, encouraging initiative, explaining the rationale behind tasks, and avoiding excessive control (Collie and Carroll, 2023), remains underexamined in hospitality.

Grounded in Self-Determination Theory (SDT; Deci and Ryan, 2000), this study proposes that ASL enhances PWB by satisfying employees' basic psychological needs: autonomy, competence, and relatedness. Psychological need satisfaction (PNS) promotes vitality and emotional balance, whereas need frustration leads to strain and impaired functioning (Gómez-Baya et al., 2018; Ryan and Deci, 2000). Despite its central role in SDT, PNS has rarely been examined as a mediating mechanism in hospitality leadership research, which often treats wellbeing as a direct outcome of leadership.

Beyond leadership behaviors, employees' experiences are shaped by the organizational context. Perceived organizational support (POS), employees' belief that their organization values their contributions and cares about their wellbeing (Eisenberger et al., 1986), may strengthen or weaken the effectiveness of ASL. While POS has been studied independently, few hospitality studies examine its interactive effects with leadership on employee outcomes (Elshaer et al., 2024; Wang, 2022).

To address these gaps, this study develops and tests a moderated mediation model, integrating SDT, Organizational Support Theory (OST), and the Stimulus–Organism–Response (S–O–R) framework (Mehrabian and Russell, 1974). Specifically, the study aims to: (1) examine the direct effects of ASL on hospitality employees' PNS and PWB, (2) investigate the mediating role of PNS, explaining how ASL translates into improved PWB, and (3) explore the moderating role of POS on both the direct and indirect relationships between ASL, PNS, and PWB.

Aligned with the objectives, the study addresses the following research questions:

1. How does ASL influence employees' PWB in hospitality settings?
2. How does ASL affect employees' PNS?
3. To what extent does PNS mediate the relationship between ASL and PWB?
4. Does POS moderate the direct relationship between ASL and PWB?
5. Does POS moderate the direct relationship between ASL and PNS?
6. Does POS moderate the indirect relationship between ASL and PWB via PNS?

By addressing these gaps, the study makes three key contributions. First, introduces ASL as a psychologically grounded approach to enhance employee wellbeing in hospitality. Second, demonstrates PNS as a mediating mechanism, advancing SDT and S–O–R applications in hospitality research. Third, highlights POS as a key contextual factor, offering practical guidance for organizations to foster healthier and more sustainable work environments.

## 2 Theoretical background and hypotheses development

### 2.1 The relationship between autonomy-supportive leadership and psychological wellbeing

ASL is a leadership style characterized by behaviors that enhance employees' autonomy, self-initiation, and intrinsic motivation. Such leaders acknowledge employees' perspectives, encourage participation and input, provide meaningful rationales for tasks, offer opportunities for choice, and minimize external control or pressure (Collie and Carroll, 2023; Shih et al., 2022). By fostering these behaviors, ASL creates a work environment that supports employees' basic psychological needs and promotes self-determined motivation, which is essential for PWB (Chang et al., 2022; Ye et al., 2025).

Empirical evidence across organizational and service contexts indicates that ASL is positively associated with employee PWB. Employees perceiving their leaders as autonomy-supportive report higher vitality, positive affect, and emotional balance, along with lower stress, emotional exhaustion, and psychological distress (Collie, 2025; Manuoglu, 2023; Ye et al., 2025). For instance, research in telework settings shows that ASL enhances both employee performance and life satisfaction, supporting the perspective that employee wellbeing and performance are mutually reinforcing (Tóth et al., 2025). Similarly, autonomy support is linked to reduced role ambiguity, role overload, and psychological distress, as well as higher subjective wellbeing. These effects have been observed across diverse contexts—including education, sports, and parenting—highlighting the broad applicability of ASL in promoting wellbeing (Slemp et al., 2018).

Despite these insights, critical evaluation reveals several limitations. First, most studies are conducted outside hospitality, leaving unclear whether ASL operates similarly under the high emotional and service demands typical of this sector. Second, prior research often reports direct effects on wellbeing without examining underlying mechanisms, limiting theoretical understanding. Third, contextual factors such as organizational support remain largely unexplored, leaving open questions about the conditions under which ASL is most effective.

From a SDT perspective, ASL fosters employees' satisfaction of three core psychological needs—autonomy, competence, and relatedness—which are fundamental for PWB (Deci and Ryan, 2000). By reducing external pressure and controlling behaviors, ASL enables employees to experience volition and mastery while enhancing social connection and trust. However, hospitality research rarely tests these mechanisms, highlighting the need for context-specific investigation. Building on SDT and prior empirical insights, this study proposes the following hypothesis:

*H1: Autonomy-supportive leadership is positively related to employees' psychological wellbeing in the hospitality industry*.

### 2.2 The relationship between autonomy-supportive leadership and psychological need satisfaction

PNS refers to the fulfillment of three basic psychological needs—autonomy, competence, and relatedness—as described in SDT (Deci and Ryan, 2000; Deci et al., 2017). Empirical research across educational, volunteer, sport, and organizational contexts consistently shows that ASL is positively associated with PNS. Leaders who acknowledge employees' perspectives, encourage self-direction, provide meaningful rationales, and minimize controlling behaviors strengthen employees' experiences of autonomy, competence, and relatedness, which in turn enhance motivation, engagement, job satisfaction, and wellbeing (Ogunmokun et al., 2022; van Tuin et al., 2020, 2025).

Evidence from diverse contexts supports this relationship. In educational settings, autonomy-supportive teaching practices increase students' satisfaction of basic psychological needs, leading to higher motivation, wellbeing, and academic achievement (Li et al., 2019). In sports, Álvarez et al. (2013) found that autonomy-supportive coaching among 159 elite taekwondo athletes enhanced need satisfaction, improving performance and PWB. Similarly, volunteer organizations demonstrate that leadership behaviors emphasizing perspective-taking and self-direction effectively foster psychological needs across different settings (Oostlander et al., 2014).

Despite this robust evidence, research on the relationship between ASL and PNS in hospitality settings remains limited. Hospitality employees often work under strict service rules, high customer demands, and constrained decision-making authority—conditions that may impede the satisfaction of basic psychological needs (Abdou et al., 2022, 2024). In such contexts, ASL may be especially critical for supporting employees' autonomy, competence, and relatedness. Accordingly, building on SDT and prior empirical findings, the present study proposes that:

*H2: Autonomy-supportive leadership is positively related to basic psychological need satisfaction among hospitality employees*.

### 2.3 The relationship between psychological need satisfaction and psychological wellbeing

SDT identifies three basic psychological needs—autonomy, competence, and relatedness—as essential for psychological growth and wellbeing (Ryan and Deci, 2000). The fulfillment of these needs is a dynamic process, shaped by both personal experiences and workplace conditions, and is fundamental to subjective and PWB across diverse contexts, including work settings (Tafvelin et al., 2019; Gil-Flórez et al., 2022). When these needs are satisfied, individuals experience higher levels of vitality, emotional balance, and psychological flexibility; conversely, frustration of these needs is associated with stress, anxiety, and depressive symptoms (Gil-Flórez et al., 2022; Peng et al., 2022).

SDT further proposes that PNS fosters intrinsic motivation, which in turn promotes positive work attitudes, engagement, and performance while reducing burnout and turnover intentions (Nunes et al., 2024; Ryan and Deci, 2000; Hooff and De Pater, 2019). Empirical studies across life and work domains consistently support this perspective, demonstrating that employees who experience higher satisfaction of autonomy, competence, and relatedness report greater life satisfaction, positive affect, vitality, and emotional balance, alongside lower levels of stress and depressive symptoms (Grinberg et al., 2025; Hood and Patton, 2022; Magson et al., 2024; Gil-Flórez et al., 2022).

Despite this robust evidence, research specifically examining the role of PNS in hospitality contexts remains limited. Hospitality employees face unique challenges, including high emotional demands, rigid procedures, and intense customer expectations, that may impede PNS and, consequently, PWB. This highlights the critical importance of testing PNS as a predictor of employees' wellbeing in service-intensive industries. Accordingly, the following hypothesis is proposed:

*H3: Psychological need satisfaction is positively related to psychological wellbeing among hospitality employees*.

### 2.4 The mediating role of psychological need satisfaction

The present study is grounded in SDT (Deci and Ryan, 2000; Deci et al., 2017) and further structured using the S–O–R model (Mehrabian and Russell, 1974) to explain how and why ASL influences employees' PWB.

First, SDT provides a motivational framework, positing that individuals' PWB is shaped by the extent to which their social and work environments satisfy three basic psychological needs: autonomy, competence, and relatedness (Deci and Ryan, 2000; Deci et al., 2017). In organizational contexts, ASL—characterized by acknowledging employees' perspectives, encouraging initiative, providing meaningful rationales, and minimizing controlling behaviors—aligns closely with these principles (Collie and Carroll, 2023; Shih et al., 2022). By fostering a supportive and non-controlling work environment, autonomy-supportive leaders enhance employees' satisfaction of these three psychological needs (Ogunmokun et al., 2022; van Tuin et al., 2020, 2025), which in turn strengthens PWB (Gil-Flórez et al., 2022; Peng et al., 2022). SDT thus offers a robust explanatory framework for understanding the motivational and psychological mechanisms through which leadership behaviors impact employee wellbeing.

Second, to complement SDT's explanatory power, the study adopts the S–O–R model as a process-oriented framework. The S–O–R model posits that environmental stimuli influence individuals' internal psychological states (organism), which subsequently shape emotional and behavioral responses. Within this study, ASL functions as the stimulus (S), PNS represents the organism (O), and PWB constitutes the response (R). This conceptualization highlights how leadership behaviors trigger internal psychological states that ultimately affect employees' overall functioning and emotional health.

Despite this theoretical foundation, empirical research has rarely tested this mediated pathway in hospitality settings, leaving a critical gap in understanding the mechanisms through which ASL enhances employee wellbeing. Accordingly, the present study proposes that.

*H4: Psychological need satisfaction has a significant mediating effect on the relationship between autonomy-supportive leadership and employees' psychological wellbeing in the hospitality sector context*.

### 2.5 The moderating role of perceived organizational support

POS refers to employees' general perception of the extent to which their organization values their contributions and cares for their wellbeing (Eisenberger et al., 1986, p. 501). In service-intensive industries such as hospitality, where employees are particularly sensitive to signals of organizational care and fairness, POS plays a critical role in shaping psychological outcomes. High POS has been linked to increased PWB, job satisfaction, engagement, and performance, as well as reduced stress, burnout, and turnover intentions (Wang et al., 2020; Bai et al., 2023; Asghar et al., 2021). Moreover, POS fosters trust in the organization and strengthens employees' psychological resources, including hope, resilience, optimism, and self-efficacy (Moustafa et al., 2024; Tang et al., 2023).

Despite its theoretical importance, research in hospitality has largely examined leadership and POS as separate predictors, with limited attention to their potential interactive effects. To the best of the author's knowledge, no previous studies have tested whether POS amplifies or conditions the effects of ASL on employees' PNS and wellbeing, leaving an important gap in understanding the boundary conditions of leadership effectiveness in high-demand service settings.

The present study draws on OST (Eisenberger et al., 1986) to address this gap. OST posits that when employees perceive organizational care and recognition, they are more likely to experience positive psychological outcomes and to be buffered against the demands of stressful work environments (Eisenberger et al., 2020). From this perspective, ASL represents direct interpersonal support, whereas POS reflects broader organizational support. When POS is high, leadership behaviors are reinforced by organizational practices, increasing their credibility and effectiveness. Consequently, the positive effects of ASL on employees' PNS and wellbeing, are likely to be strengthened.

Empirical evidence outside hospitality supports this moderating role: POS has been shown to buffer the negative effects of despotic or exploitative leadership on employee wellbeing (Mehmood et al., 2023; Wang et al., 2021). Similarly, high POS may strengthen the positive effects of ASL by creating a supportive environment where leadership behaviors more effectively satisfy employees' autonomy, competence, and relatedness (Hejazi et al., 2025). Conversely, low POS may weaken these effects, reducing the benefits of ASL (Kong et al., 2026).

Building on OST and SDT, POS is also expected to moderate the indirect relationship between ASL and PWB through PNS. When POS is high, organizational policies, practices, and resources reinforce leaders' autonomy-supportive behaviors, strengthening employees' satisfaction of basic psychological needs and amplifying the positive effect on wellbeing. Thus, POS functions as a critical boundary condition influencing both the direct and mediated effects of ASL in hospitality settings. Hence, the following hypotheses are suggested:

*H5: Perceived organizational support moderates the relationship between autonomy-supportive leadership and employees' psychological wellbeing, such that the relationship is stronger when perceived organizational support is high*.
*H6: Perceived organizational support moderates the relationship between autonomy-supportive leadership and psychological need satisfaction, such that the relationship is stronger when perceived organizational support is high*.
*H7: Perceived organizational support moderates the indirect relationship between autonomy-supportive leadership and employees' psychological wellbeing through psychological need satisfaction, such that the indirect effect is stronger when perceived organizational support is high*.

**Figure 1:**
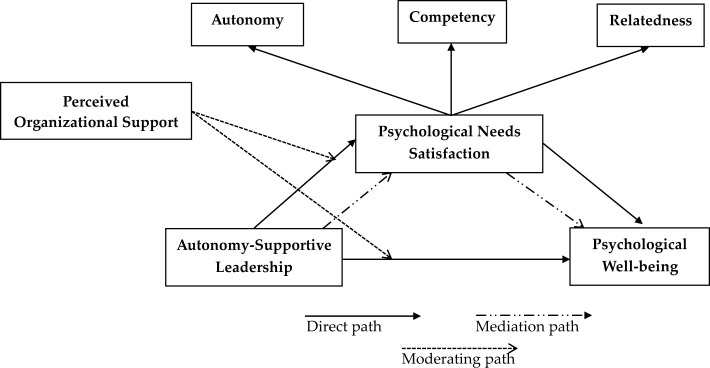
Study's conceptual framework.

Figure 1 illustrates the study's conceptual framework, integrating ASL, PNS, PWB, and POS.

## 3 Methodology

### 3.1 Sampling and data collection

This study employed a non-probability purposive sampling technique, which is appropriate when participants are selected based on predefined criteria aligned with the research objectives (Lavrakas, 2008). This approach is commonly used in hospitality and leadership research to ensure that respondents possess sufficient experience to meaningfully evaluate leadership behaviors and psychological outcomes (Darvishmotevali and Altinay, 2022; Abdou, 2025). However, consistent with methodological recommendations, the use of purposive sampling may introduce selection bias and limit the generalizability of findings beyond the studied context. Therefore, the results should be interpreted with caution, particularly when extending conclusions to other sectors or hospitality settings with different characteristics.

To enhance the relevance and validity of responses, three selection criteria were applied. First, participants were required to be front-line employees (FLEs) who regularly interact with customers (e.g., front desk agents, food and beverage staff, housekeeping, and guest service employees), as these roles are directly influenced by leadership behaviors and are critical to service delivery. Second, only full-time employees were included to ensure sufficient and consistent interaction with supervisors. Third, participants were required to have a minimum of 1 year of tenure, ensuring adequate exposure to organizational practices and leadership styles. These criteria were designed to strengthen the internal validity of the study by targeting respondents capable of providing informed evaluations.

Based on these criteria, data were collected from FLEs working in 12 five-star hotels in the Kingdom of Saudi Arabia (KSA). The participating hotels—five in Makkah, four in Jeddah, and three in Riyadh—represent major hospitality hubs characterized by high service intensity and emotional labor demands (Abolnasser et al., 2023; Abdou, 2025). While focusing on five-star hotels enhances contextual consistency and relevance for examining leadership and wellbeing in high-contact service environments, it may also limit external validity, as findings may not fully generalize to lower-tier hotels or other service sectors.

Before data collection, permission was obtained from human resource managers in the participating hotels. To reduce common method bias (CMB), data were collected using a time-lagged design across three waves, each separated by a 2 week interval. Questionnaires were self-administered during employees' break periods and coordinated through designated hotel representatives. Participation was voluntary, and informed consent was obtained from all respondents. Anonymity and confidentiality were assured, and completed questionnaires were returned in sealed envelopes to minimize social desirability and response bias, ensuring compliance with ethical research standards.

To match responses across the three waves, participants created a unique self-identification code (last four digits of their phone number), which was used on all questionnaires to preserve anonymity. At Time 1 (T1), employees reported their perceptions of ASL and provided demographic information. A total of 600 forms were distributed, and only 501 (83.5%) were received. At Time 2 (T2), conducted 2 weeks after T1, 501 questionnaires, measuring POS and PNS, were distributed to the respondents who participated in T1. Of these, 441 (88%) usable questionnaires were returned. At Time 3, 2 weeks after T2, 441 questionnaires measuring PWB were distributed to the remaining participants. A total of 394 (89.3%) usable questionnaires were returned and included in the final analysis. Table 1 presents the demographic characteristics of the participants.

**Table 1:** Demographic characteristics of the participants.

Demographic variables	No.	%
Gender	
Male	222	56.35
Female	172	43.65
Age (years)	
18–25	161	40.86
26–35	181	45.94
36–45	38	9.64
More than 45	14	3.56
Educational level	
High school degree or equivalent	97	24.62
University degree	265	67.26
Postgraduate degree	32	8.12
Experience within the hotel (years)	
1–3	157	39.85
3–5	173	43.91
5–10	42	10.66
More than 10	22	5.58
Total	394	100%

### 3.2 Instrument development and measures of the study

The self-administered questionnaire used in this study was derived from well-established and previously validated scales to ensure content adequacy and theoretical consistency. All measurement items were carefully adapted to the hospitality context while preserving their original conceptual meaning. To strengthen content validity, a multi-stage validation process was employed. First, the initial instrument was reviewed by four academic experts in hospitality management and organizational behavior. Their feedback led to refinements in item wording, improved clarity, and ensured alignment between the items and the study constructs. Second, a pilot test was conducted with 30 front-line hotel employees to assess readability and comprehension, resulting in minor modifications to enhance clarity and contextual relevance.

Given the multilingual research context, the questionnaire was administered in both English and Arabic. To ensure linguistic equivalence, a back-translation procedure was applied (Cruchinho et al., 2024). The original English version was translated into Arabic by a bilingual expert and then independently back-translated into English by another translator. The comparison of the two versions revealed no significant discrepancies, supporting translation accuracy.

The final questionnaire consisted of five sections. The first section collected respondents' demographic information. The remaining sections measured the study constructs using established scales. ASL was measured using a five-item scale adapted from Collie (2021) that captures leadership behaviors such as acknowledging employee perspectives and providing choice (e.g., “My manager provides me with a choice for how I go about my work”). The Cronbach's alpha coefficient for this measure was 0.898.

PNS was assessed using the Need Satisfaction at Work Scale (NSa-WS) developed by Tafvelin and Stenling (2018). Consistent with SDT, PNS was modeled as a second-order construct comprising 13 items across three dimensions: autonomy (four items), competence (four items), and relatedness (five items). Selected items from the scale include “I can make meaningful choices in my work,” “I can handle the challenges I face in my job,” and “I feel close to the people I work with.” The Cronbach's alpha coefficient for this measure was 0.936.

PWB was measured using a 10-item scale adapted from Pradhan and Hati (2022) and previously applied in hospitality research (Abolnasser et al., 2023). A sample item is “I feel psychologically comfortable and able to cope with the demands of daily life.” The Cronbach's alpha coefficient for this measure was 0.903.

POS was assessed using a six-item scale adapted from Eisenberger et al. (1986) that captures employees' perceptions of organizational care and support (e.g., “My hotel really cares about my wellbeing”). The Cronbach's alpha coefficient for this measure was 0.941. All responses were recorded on a five-point Likert scale ranging from “1 = strongly disagree to 5 = strongly agree.”

### 3.3 Data analysis

To ensure methodological rigor, this study adopted a two-step analytical approach in line with established recommendations (Kline, 2023; Hair et al., 2019). In the first step, the measurement model was assessed to verify the psychometric adequacy of all constructs. Specifically, internal consistency reliability was evaluated using Cronbach's alpha and composite reliability, while convergent validity was assessed through indicator loadings and average variance extracted (AVE). In addition, discriminant validity was examined using both the Fornell–Larcker criterion and the heterotrait–monotrait ratio (HTMT). This step ensured that all constructs demonstrated sufficient reliability and validity before testing the structural relationships.

In the second step, the study employed Partial Least Squares Structural Equation Modeling (PLS-SEM) using SmartPLS (v. 4.1.1.4) to test the hypothesized relationships. The use of PLS-SEM is particularly appropriate for this study for several reasons. First, our study is primarily prediction-oriented and involves a relatively complex model incorporating mediating (PNS) and moderating (POS) effects, which require simultaneous estimation of multiple relationships. Second, PLS-SEM is well-suited for prediction-oriented research and theory development, aligning with the study's objective of explaining psychological mechanisms in the hospitality context. Third, this approach is robust to non-normal data distributions and is appropriate for applied field data, which often deviates from normality.

To assess the significance of the structural paths, including direct, indirect (mediation), and interaction (moderation) effects, a bootstrapping procedure with 5,000 resamples was conducted to generate confidence intervals and *t*-values. Furthermore, model evaluation criteria, including path coefficients, explained variance (*R*^2^), and predictive relevance, were applied to assess the overall explanatory power of the model.

## 4 Results

### 4.1 Measurement model assessment

The measurement model was evaluated in accordance with the recommendations of Hair et al. (2019). Four steps were adopted. Indicator reliability was assessed by examining outer loadings, with values above 0.708 considered acceptable. Table 2 shows that all standardized factor loadings (SFLs) are significant and exceed 0.708. Internal consistency reliability was assessed using Cronbach's alpha and composite reliability (CR). Thresholds of 0.70 or higher indicate satisfactory reliability. The results in Table 2 confirm that all study constructs exhibit satisfactory internal consistency reliability. Furthermore, all constructs achieved adequate convergent validity, as evidenced by AVE-values ≥ 0.50.

**Table 2:** Measurement model analysis.

Construct	Item	SFL	Alpha (α)	CR	AVE
Autonomy-supportive leadership (ASL)	ASL1	0.759^***^	0.898	0.926	0.714
	ASL2	0.775^***^			
	ASL3	0.900^***^			
	ASL4	0.883^***^			
	ASL5	0.897^***^			
**Psychological need satisfaction (PNS)**			0.936	0.944	0.564
Autonomy	PNS1	0.756^***^	0.778	0.858	0.601
	PNS2	0.788^***^			
	PNS3	0.785^***^			
	PNS4	0.771^***^			
Competency	PNS5	0.767^***^	0.740	0.837	0.562
	PNS6	0.723^***^			
	PNS7	0.758^***^			
	PNS8	0.751^***^			
Relatedness	PNS9	0.718^***^	0.814	0.877	0.590
	PNS10	0.723^***^			
	PNS11	0.832^***^			
	PNS12	0.712^***^			
	PNS13	0.844^***^			
**Psychological wellbeing (PWB)**	PWB1	0.735^***^	0.903	0.924	0.552
	PWB2	0.789^***^			
	PWB3	0.780^***^			
	PWB4	0.714^***^			
	PWB5	0.765^***^			
	PWB6	0.757^***^			
	PWB7	0.715^***^			
	PWB8	0.742^***^			
	PWB9	0.709^***^			
	PWB10	0.719^***^			
**Perceived organizational support (POS)**	POS1	0.938^***^	0.941	0.972	0.854
	POS2	0.931^***^			
	POS3	0.921^***^			
	POS4	0.931^***^			
	POS5	0.916^***^			
	POS6	0.909^***^			

SFL, standardized factor loading; CR, composite reliability; AVE, average variance extracted.

^***^p <0.001.

Beyond reliability and convergent validity, discriminant validity (DV) was evaluated using the Fornell–Larcker criterion together with the HTMT ratio. According to the Fornell–Larcker criterion, √AVE > inter-construct correlations supports construct discriminant validity (Fornell and Larcker, 1981). On the other side, concerning the HTMT ratio, values above the threshold (0.90) indicate a lack of discriminant validity (Henseler et al., 2015). Based on these criteria, the results in Table 3 confirm construct distinctiveness.

**Table 3:** The discriminant validity.

Construct	1	2	3	4
HTMT ratio				
1. Autonomy-supportive leadership				
2. Perceived organizational support	0.796			
3. Psychological need satisfaction	0.643	0.536		
4. Psychological wellbeing	0.787	0.631	0.641	
Fornell–Larcker criterion				
1. Autonomy-supportive leadership	**0.845**			
2. Perceived organizational support	0.742	**0.924**		
3. Psychological need satisfaction	0.590	0.511	**0.751**	
4. Psychological wellbeing	0.731	0.635	0.606	**0.743**

All HTMT values are lower than 0.90.

Bold diagonal values are √AVE.

### 4.2 Descriptive statistics

Table 4 presents the descriptive statistics and latent variable correlations among the study constructs. The mean values range from 3.71 to 3.96, indicating moderate to high levels of the focal variables. PNS reported the highest means (*M* = 3.96, SD = 1.34), followed by POS, PWB, and ASL, respectively. The standard deviations suggest adequate variability across constructs.

**Table 4:** Descriptive statistics and correlations between the study's variables.

Construct	Mean	Standard deviation	1	2	3	4
Autonomy-supportive leadership	3.71	1.44	1			
Perceived organizational support	3.82	1.35	0.742^C^,^***^	1		
Psychological need satisfaction	3.96	1.34	0.590^C^,^***^	0.511^C^,^***^	1	
Psychological wellbeing	3.75	1.48	0.731^C^, ^***^	0.635^C^, ^***^	0.606^C^, ^***^	1

^*C*^Latent variables correlation.

^***^p < 0.001.

Regarding associations, all latent variable correlations are positive and statistically significant (*p* < 0.001). ASL is significantly correlated with POS (*r* = 0.742), PWB (*r* = 0.731), and PNS (*r* = 0.590). POS is significantly positively associated with PNS (*r* = 0.511) and PWB (*r* = 0.635). PNS also shows a significant positive correlation with PWB (*r* = 0.606). Overall, these results provide preliminary support for the hypothesized relationships among the study variables.

### 4.3 Collinearity analysis

Collinearity was assessed before evaluating the structural model to ensure that the estimated path coefficients were not biased by multicollinearity issues. Following the recommendations of Kock (2015), variance inflation factor (VIF) values were examined for all predictor constructs. The results indicate that all VIF values were below the conservative threshold of 3.3, suggesting that collinearity is not a concern in the present study (see Table 5). Therefore, the structural model estimates can be interpreted with confidence.

**Table 5:** Collinearity statistics via VIF.

Constructs	Psychological need satisfaction	Psychological wellbeing
Autonomy-supportive leadership	2.243	2.572
Perceived organizational support	2.596	2.676
Psychological need satisfaction		1.588
POS × ASL	1.263	1.284

### 4.4 Structural model assessment

**Figure 2:**
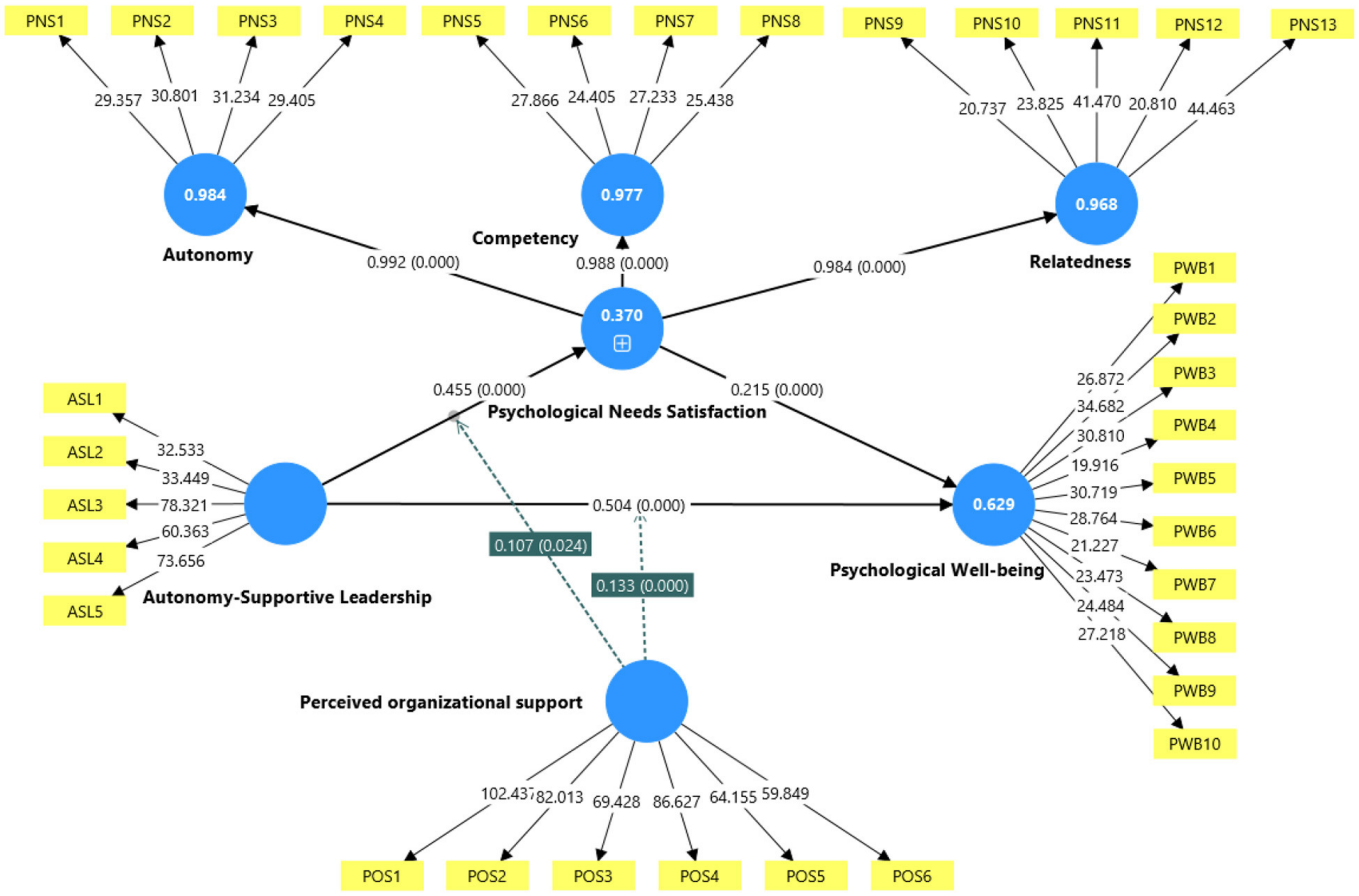
The structural model.

After establishing the adequacy of the measurement model, the structural model was evaluated using PLS-SEM. In addition to statistical significance, the analysis emphasizes effect sizes, explanatory power, and predictive relevance to provide a more comprehensive understanding of the results. The hypothesized relationships were tested using a bootstrapping procedure with 5,000 resamples. As shown in Tables 6, 7 and illustrated in Figure 2, all direct and indirect relationships are positive and statistically significant.

**Table 6:** Estimates of structural parameters.

Hypothesized path	Original sample (O)	Sample mean (M)	Standard deviation (STDEV)	*T* statistics	*f* ^2^	Confidence intervals		Results
						2.5%	97.5%	
H_1_: ASL –> PWB	0.504	0.508	0.041	12.336^***^	0.267	0.428	0.589	Supported
H_2_: ASL –> PNS	0.455	0.459	0.059	7.742^***^	0.146	0.344	0.575	Supported
H_3_: PNS –> PWB	0.215	0.214	0.045	4.829^***^	0.079	0.127	0.301	Supported
Mediating path								
H_4_: ASL –> PNS –> PWB	0.098	0.098	0.024	4.002^***^		0.054	0.149	Supported
Moderating path								
H_5_: POS × ASL –> PWB	0.133	0.133	0.032	4.106^***^	0.042	0.07	0.196	Supported
H_6_: POS × ASL –> PNS	0.107	0.105	0.047	2.260^*^	0.026	0.012	0.201	Supported
Moderated-mediation path								
H_7_: POS × ASL –> PNS –> PWB	0.023	0.022	0.011	2.181^*^		0.003	0.045	Supported

ASL, autonomy-supportive leadership; PWB, psychological wellbeing; PNS, psychological need satisfaction; POS, perceived organizational support.

^*^p <0.05,

^***^p <0.001.

**Table 7:** *R*^2^ values and predictive relevance (*Q*^2^predict) of the model.

Constructs	*R* ^2^	*Q* ^2^_predict_
Psychological need satisfaction	0.370	0.352
Psychological wellbeing	0.626	0.579

First, the results support H1, demonstrating that ASL is positively associated with employees' PWB in the hospitality industry context (β = 0.504, *t*-value = 12.336, *p* < 0.001, and *f*^2^= 0.267). Based on Cohen's (1988) guidelines, where values of 0.02, 0.15, and 0.35 indicate small, medium, and large effects, respectively, these results illustrate that ASL exerts a medium-to-large effect on PWB.

Second, supporting Hypothesis 2, ASL is positively associated with employees' PNS. As shown in Table 6, the relationship is statistically significant (β = 0.455, *t* = 7.742, *p* < 0.001, and *f*^2^ = 0.146). Based on Cohen's benchmarks, this reflects a moderate effect size, indicating that ASL plays a meaningful and substantive role in fulfilling employees' basic psychological needs. At the same time, the magnitude of the effect suggests that PNS is likely influenced by additional organizational and contextual factors beyond leadership alone. Third, Hypothesis 3 is also supported, as PNS exhibits a significant positive relationship with PWB (β = 0.215, *t* = 4.829, *p* < 0.001, and *f*^2^ = 0.079). According to Cohen's benchmarks, this represents a small to medium effect size, indicating that while PNS contributes meaningfully to employees' wellbeing, its practical impact is relatively modest. This suggests that PWB is shaped by multiple factors and that mechanisms beyond need satisfaction also play an important role in explaining employees' overall wellbeing.

Fourth, regarding the indirect effect, Hypothesis 4 proposed that PNS mediates the relationship between ASL and PWB. As shown in Table 6, the indirect effect is positive and statistically significant (β = 0.098, *t* = 4.002, and *p* < 0.001), with the confidence interval [0.054, 0.149] excluding zero, thereby confirming the robustness of the mediation effect. Consistent with the mediation framework of Zhao et al. (2010), PNS functions as a partial mediator, suggesting that ASL influences PWB both directly and indirectly through the fulfillment of employees' basic psychological needs.

**Figure 3:**
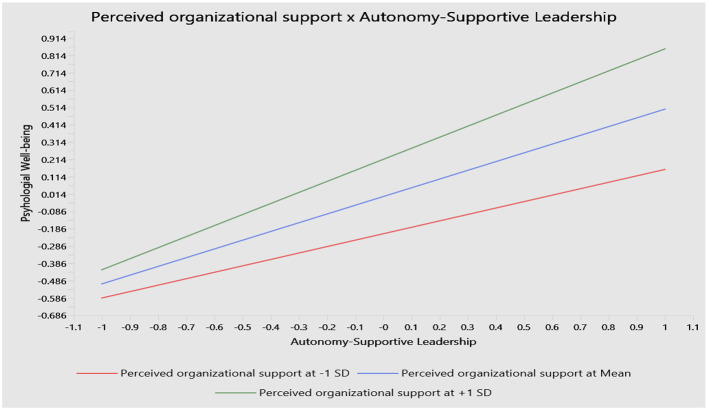
The moderating role of POS in the association between ASL and PWB.

The moderating effects of POS on the relationships between ASL and the outcome variables were examined to test Hypotheses 5, 6, and 7. Results from path analysis illustrate that POS significantly moderates the relationship between ASL and PWB (H5). The interaction effect (POS × ASL) on PWB is positive and statistically significant (β = 0.133, *t* = 4.106, and *p* < 0.001), with a confidence interval of [0.07, 0.196], supporting Hypothesis 5. The simple slope results show that the positive relationship between ASL and PWB is strengthened at higher levels of POS (+1 SD), whereas the relationship is weaker when POS is low (−1 SD; see Figure 3).

**Figure 4:**
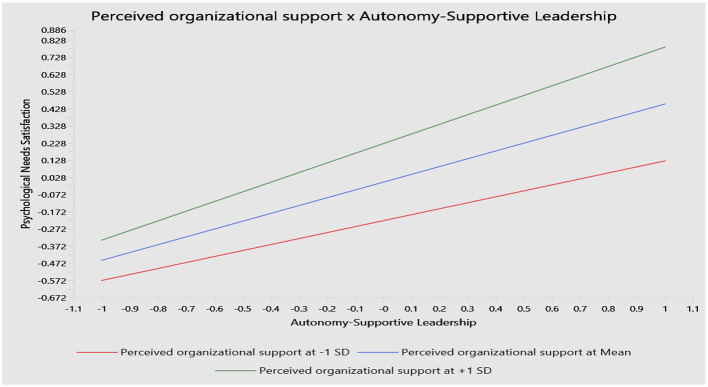
The moderating role of POS in the association between ASL and PNS.

Similarly, Hypothesis 6, which proposed a moderating role of POS in the relationship between ASL and PNS, is also supported. The interaction term (POS × ASL) exhibits a significant positive effect on PNS (β = 0.107, *t* = 2.260, and *p* < 0.05), with a confidence interval of [0.012, 0.201]. The simple slope analysis indicates that the positive relationship between ASL and PNS is stronger at higher levels of POS (+1 SD) and weaker when POS is low (−1 SD; see Figure 4).

The bootstrapped conditional indirect effect results obtained from PLS-SEM also provide support for Hypothesis 7, confirming that POS significantly moderates the indirect relationship between ASL and PWB via PNS. Specifically, the interaction term (ASL × POS) shows a significant positive indirect effect on PWB through PNS (β = 0.023, *t* = 2.181, and *p* < 0.05). The bias-corrected 95% bootstrapped confidence interval does not include zero [0.003, 0.045], confirming the presence of a significant moderated mediation effect.

Results demonstrate that, although the moderating effects of POS are statistically significant, their effect sizes are relatively small. Specifically, the interaction effects on PWB (*f*^2^ = 0.042) and PNS (*f*^2^ = 0.026) fall within Cohen's small effect threshold, indicating limited practical magnitude. Similarly, the moderated mediation effect (β = 0.023) is small in size, suggesting that the conditional indirect effect contributes only modestly to the overall model. Overall, these findings indicate that the influence of POS is incremental rather than substantial.

Beyond path coefficients, the model's explanatory power was assessed using *R*^2^ values. The results show that ASL and POS explain 37.0% of the variance in PNS, while the full model explains 62.6% of the variance in PWB, indicating moderate to substantial explanatory power. This suggests that the model captures a meaningful proportion of employees' psychological outcomes. Furthermore, predictive relevance was examined using *Q*^2^predict. The obtained values (***Q***^2^_**predict**_ = 0.352 for PNS and ***Q***^2^_**predict**_ = 0.579 for PWB) exceed zero, indicating strong predictive capability and confirming that the model performs well in predicting out-of-sample observations.

### 4.5 Control variables

We have used demographic variables (age, gender, education level, and tenure) as control variables in our analysis. The variables were included to determine the potential impact of these variables on the relationships between ASL and its related outcomes (e.g., PNS and PWB). The results, however, indicate that demographic variables have not significantly impacted the model relationships. This implies that the essential effects of employees' PNS and wellbeing remain valid regardless of demographic differences.

## 5 Discussion and implications

### 5.1 Key findings

The present study was designed to empirically evaluate a moderated-mediation model exploring the interactive roles of ASL, psychological needs satisfaction, and POS in predicting PWB among hospitality employees. This approach allowed us to investigate both the direct and indirect effects of ASL on employees' wellbeing, as well as the moderating role of POS in these relationships. The findings provided support for all of the study's hypotheses. Several important insights emerge from these findings, highlighting the mechanisms through which leadership and organizational support jointly influence employees' psychological outcomes in the hospitality context.

First, the findings of this study provide empirical support for the proposed positive relationship between ASL and employees' PWB in the hospitality industry. Consistent with Hypothesis 1, ASL had a significant positive association with PWB, indicating that employees who perceive their leaders as autonomy-supportive experience higher levels of PWB. This result aligns closely with SDT (Deci and Ryan, 2000), which posits that work environments that foster autonomy, competence, and relatedness facilitate optimal psychological functioning and wellbeing. The present findings are also consistent with prior empirical research showing that ASL is associated with higher vitality, positive affect, and emotional balance, as well as lower psychological distress and emotional exhaustion (Collie, 2025; Manuoglu, 2023; Ye et al., 2025; Tóth et al., 2025). Extending prior literature, this study shows that autonomy-supportive leaders foster sustainable employee wellbeing in the hospitality industry by acknowledging employees' perspectives, encouraging participation, and providing meaningful rationales for work tasks.

Second, the findings of this study further confirm a positive relationship between ASL and employees' PNS in the hospitality industry. Consistent with SDT (Deci and Ryan, 2000), this result suggests that when leaders listen to employees, encourage them to take initiative, and avoid overly controlling behavior, employees are more likely to feel autonomous, capable, and connected at work. These findings align with prior research conducted in educational, sport, volunteer, and organizational contexts, which demonstrates that ASL promotes PNS and, in turn, positive motivational and wellbeing outcomes (Ogunmokun et al., 2022; van Tuin et al., 2020, 2025). By focusing on the hospitality sector, this study fills an important gap in the literature by demonstrating that ASL remains a vital resource, even in highly structured and demanding service environments.

Third, PNS is positively associated with PWB among hospitality employees, supporting Hypothesis 3. This result aligns with the lens of SDT. The theory emphasizes that the fulfillment of the basic psychological needs, autonomy, competence, and relatedness, is fundamental for individuals' psychological growth and wellbeing (Ryan and Deci, 2000). Consistent with previous empirical research, our findings indicate that when employees experience satisfaction with these needs, they report higher levels of PWB (Gil-Flórez et al., 2022; Lataster et al., 2022; Tang et al., 2020). In contrast, the frustration of these needs is linked to negative outcomes such as stress, anxiety, and depressive symptoms (Gil-Flórez et al., 2022; Peng et al., 2022).

Fourth, PNS significantly mediated the linkage between ASL and employees' PWB in the hospitality industry. These findings align closely with the theoretical predictions of SDT and offer important insights into the mechanisms through which leadership behaviors influence employee psychological outcomes. Specifically, the partial mediation effect indicates that in the hospitality context, ASL predicts employees' PWB not only directly but also indirectly by satisfying their fundamental psychological needs for autonomy, competence, and relatedness. Leaders who acknowledge employees' perspectives, encourage initiative, provide meaningful foundations, and reduce controlling practices create a work environment that nurtures need satisfaction (Ogunmokun et al., 2022; van Tuin et al., 2020, 2025), which in turn enhances PWB. The significant mediation effect observed in this study additionally supports this S–O–R model, highlighting PNS as a crucial (organism) mechanism linking leadership behaviors to employee psychological outcomes.

Fifth, the findings of the study reveal that POS strengthens the relationship between ASL and employees' PWB. These findings are consistent with OST (Eisenberger et al., 1986), suggesting that employees who perceive high levels of organizational support are more receptive to leaders' autonomy-enhancing behaviors and, as a result, experience higher PWB. This underscores the importance of a supportive organizational climate in which leadership efforts are reinforced through practices such as recognition, fair policies, and wellbeing initiatives. These results align with prior research indicating that POS amplifies the positive effects of leadership while buffering employees against stress and workplace challenges (Mehmood et al., 2023; Wang et al., 2021). Based on this finding, it could be concluded that organizational support acts as a key boundary condition that determines the effectiveness of leadership behaviors in high-demand hospitality environments.

Sixth, the findings that POS strengthens the association between ASL and employees' PNS further reinforce the role of organizational support in shaping internal motivational processes. Consistent with OST (Eisenberger et al., 1986), employees who perceive strong organizational support experience higher levels of autonomy, competence, and relatedness when leaders engage in autonomy-supportive behaviors. This demonstrates that organizational support not only validates leadership efforts but also provides the broader context necessary for employees to fully experience PNS. These results also align with previous studies, which have shown that POS enhances employees' capacity to satisfy their psychological needs and promotes better motivational outcomes (Hejazi et al., 2025; Kong et al., 2026). In contrast, in environments with low POS, employees' psychological responses to ASL are weakened, indicating that organizational support is essential to maximize the impact of leadership on internal psychological states.

Finally, the findings support Hypothesis 7 proposed the moderating effect of POS in the indirect relationship between ASL and PWB through PNS. This result indicates that the positive indirect effect of ASL on PWB via PNS is conditional upon levels of POS. Consistent with SDT and OST, when POS is high, organizational policies, practices, and resources reinforce leaders' autonomy-supportive behaviors, thereby enhancing employees' satisfaction of autonomy, competence, and relatedness and strengthening the positive indirect effect on PWB. This finding also aligns with the S–O–R framework, whereby POS functions as a contextual boundary condition that shapes the leadership stimulus, the organismic state of PNS, and the resulting wellbeing outcomes.

### 5.2 Theoretical implications

This study offers several important theoretical implications for the literature on leadership, employee wellbeing, and motivation in the hospitality context. First, by empirically validating the positive effect of ASL on employees' PWB, this study extends SDT into the hospitality domain, where its application remains limited. Unlike previous SDT research primarily conducted in education and healthcare, our findings demonstrate that ASL acts as a critical social-contextual resource that fosters employees' wellbeing in high-demand hospitality settings. Second, the study contributes to SDT by empirically confirming the central role of PNS as a key explanatory mechanism linking leadership behaviors to PWB. This emphasizes the internal motivational processes through which leadership influences employee outcomes, moving beyond simple direct effects.

Third, by integrating SDT with the S–O–R framework, the study clarifies both motivational and process-based pathways, showing how leadership behaviors (stimuli) influence internal psychological states (organism) that, in turn, drive wellbeing responses (response). Notably, positioning PNS as the central “organism” state provides a novel conceptual contribution by highlighting the mediating role of employees' psychological processes within hospitality leadership research. Fourth, the study introduces POS as a boundary condition that shapes the effectiveness of ASL, drawing on OST. Our findings reveal that POS interacts with leadership behaviors to enhance PNS and PWB, demonstrating that organizational-level support does not operate in isolation but amplifies the effects of leadership. This integration of SDT and OST within a single framework advances understanding of contextual contingencies in leadership research.

Finally, this study contributes a novel moderated mediation model that simultaneously incorporates ASL, PNS, POS, and PWB in the hospitality context. While prior research has examined direct or simple mediation effects of leadership, the present study is among the first to integrate motivational mediators with organizational moderators to test complex conditional pathways in hospitality settings. This provides a more nuanced, theory-driven framework for understanding how leadership behaviors translate into employee wellbeing outcomes, offering a foundation for future studies to explore multilevel and context-dependent leadership processes.

In sum, the study advances SDT-based leadership research by demonstrating how ASL, mediated by PNS and moderated by POS, contributes to employee PWB in hospitality, a combination not previously tested empirically in this context.

### 5.3 Managerial implications

The findings of this study offer specific and actionable implications for hospitality managers seeking to enhance employees' PWB. Given the significant positive effect of ASL on PNS and wellbeing, managers should implement structured practices that promote autonomy in daily operations. For example, supervisors can incorporate short weekly team briefings where employees are invited to suggest service improvements or participate in decision-making related to guest interactions. Additionally, instead of enforcing rigid procedures, managers should provide clear rationales for service standards and allow frontline employees discretion in handling customer requests, particularly in high-contact roles such as front office and food and beverage services.

Furthermore, since PNS was found to mediate the relationship between leadership and wellbeing, organizations should introduce targeted interventions to strengthen autonomy, competence, and relatedness. This can be operationalized through job design strategies such as task rotation to enhance competence, structured peer-support systems or buddy programs to strengthen relatedness, and empowerment policies that allow employees to resolve guest complaints without excessive managerial approval. Regular, skills-based feedback sessions—rather than purely evaluative appraisals—can further reinforce employees' sense of competence.

In addition, the moderating role of POS suggests that leadership behaviors are more effective when reinforced by organizational practices. Therefore, hospitality organizations should align HR policies with leadership initiatives by introducing formal recognition programs (e.g., monthly acknowledgment of service excellence), transparent scheduling systems to improve fairness, and accessible wellbeing resources such as stress management workshops or counseling services. Importantly, managers should be trained not only to display supportive behaviors but also to communicate organizational support clearly, ensuring employees recognize these efforts.

Finally, to sustain these outcomes, organizations should implement continuous monitoring systems. This includes periodic employee surveys specifically measuring autonomy support, PNS, and wellbeing, as well as tracking indicators such as burnout, absenteeism, and service quality.

## 6 Study limitations and future research

Despite its contributions, this study has several limitations that should be considered when interpreting the findings and that open avenues for future research. From a methodological perspective, the use of purposive sampling and data collected from frontline employees in five-star hotels may limit external validity, as the findings may not generalize to other hospitality segments or service industries with different structural and operational characteristics. Future research is encouraged to examine diverse settings, such as mid-range or budget hotels, and to include participants from different cultural backgrounds to enhance the applicability of the results. Further, while we employed a time-lagged design, collecting all data from the same participants over a 2 week interval may limit strong causal inference and introduce concerns regarding common method bias and subjective perception. To address this limitation, future studies are encouraged to use multi-source data and longer-term longitudinal designs to enhance causal inference and reduce potential biases.

From a theoretical perspective, this study focused on PNS as the primary mediating mechanism linking ASL to PWB. However, other relevant psychological processes, such as work engagement, psychological capital, leader–member exchange, or emotional exhaustion, may also explain this relationship and should be examined in future research. Similarly, while POS was treated as a key boundary condition, additional contextual moderators (e.g., service climate, organizational justice, leadership climate, or HR practices) may further shape the effectiveness of ASL.

From a contextual standpoint, the study was conducted within the Saudi Arabian hospitality sector, which is characterized by unique cultural and organizational dynamics that may influence leadership perceptions and employee responses. Future research should replicate and extend the proposed model across different cultural settings and service industries (e.g., airlines, restaurants, or cruise lines) to enhance generalizability. Finally, future studies could broaden the scope of the model by examining additional outcomes, such as service sabotage, creativity, and customer-oriented behaviors, and by testing alternative model specifications to further strengthen the explanatory power and robustness of the proposed framework.

## 7 Conclusion

This study advances understanding of employee wellbeing in hospitality by demonstrating that ASL operates as a key contextual resource that enhances PWB both directly and indirectly through PNS. By integrating SDT with the S–O–R framework, the findings clarify that leadership influences wellbeing not only as an external stimulus but also through internal psychological mechanisms, positioning need satisfaction as a central explanatory pathway. Furthermore, the results extend OST by showing that POS acts as a critical boundary condition that amplifies both the direct and indirect effects of leadership. Collectively, the study contributes a theoretically integrated model that explains how leadership and organizational context jointly shape employee wellbeing in high-demand service environments, offering a more nuanced and process-based understanding of these relationships.

## Data Availability

The original contributions presented in the study are included in the article/supplementary material, further inquiries can be directed to the corresponding author.
